# The impact of China's urban and rural economic revitalization on the utilization of mental health inpatient services

**DOI:** 10.3389/fpubh.2022.1043666

**Published:** 2023-01-12

**Authors:** Yu Yan, Yongqian Tu

**Affiliations:** ^1^School of Law, Guangdong University of Technology, Guangzhou, Guangdong, China; ^2^National Academy of Development and Strategies, Renmin University of China, Beijing, China

**Keywords:** economic burden, frequency of hospitalization, health insurance policy, hospitalization costs, length of hospitalization, mental health, reimbursement ratio

## Abstract

**Background:**

Rural locations have a lower preference for mental healthcare than urban areas. Medical and pharmacy expenses incurred as a result of serious mental illness are covered by public health insurance, according to the People's Republic of China's Mental Health Law. This study aimed to acknowledge the disparities in the use of mental health services provided by the government health schemes among the rural and urban populations of China and to assess the real reimbursement rates for health insurance coverage. It also sheds light on China's ongoing healthcare reforms for mental health treatments.

**Materials and methods:**

A retrospective cohort study of patients was conducted that were hospitalized with mental illnesses to assess rural–urban disparities in the utilization of mental health services and the role of health insurance. We used electronic health data from the major psychiatric institutes for 15 years (2005–2020) to assess the influence of health insurance systems on Chinese public preferences for mental health treatments. These psychiatric hospitals serve almost 10% of all mental health patients every year in Shandong and accept patients from all over the country. In addition, health insurance policy regulations in Shandong Province are consistent with national health insurance policy regulations. Models 1 and 2 assess disparities in the utilization of mental health treatments. Our study population was identified using patients' primary diagnosis, as recorded in the two hospitals' EHRs, which routinely record information on patients' sociodemographic characteristics, clinical characteristics of the disease, cost of the treatment, and type of the health insurance plan. The record of EHR data is considered efficient because they document all inpatient expenses incurred during hospitalization in a detailed, itemized, and reliable way.

**Results:**

Urban patients had longer hospital stays (*p* = 0.0001), more hospitalizations (*p* = 0.006), and greater hospitalization expenses (*p* = 0.001) than rural patients. Patients who had insurance had a longer hospital stay (*p* = 0.0001), more hospitalizations (*p* = 0.0001), and greater hospitalization costs (*p* = 0.0001) than those who did not have insurance. Urban residents used mental health services more than rural residents. People preferred mental healthcare when the reimbursement ratio variable was larger, especially in rural areas.

**Conclusion:**

Rural people of China experience mental health services are an economic burden. Uniform measures are required to be taken for the development of health insurance policies for people in rural areas.

## Introduction

China's economic reform and rapid urbanization over the past three decades have led to unprecedented massive rural-to-urban migration. Epidemiological research has documented that migration and urbanicity both contribute to the risks of mental illness, associated with particular features of the urban environment or difficulties encountered in migration (1, 2). Concerns about the burden of mental disorders and their association with urbanicity have grown worldwide.

Mental healthcare, on the contrary, is less favored than physical health treatments (3). Patients often choose psychiatric therapy in the latter stages of sickness, which leads to various mental and physical comorbidities and the use of several life-threatening drug(s) misuse by patients (4). Although mental health treatments are expensive in most countries, they are not covered by insurance (5). In China, for various cultural, socioeconomic, and healthcare-related reasons, people with mental health needs have long been underserved (6). The preference for mental health services is almost the same in China's urban and rural locations, however in rural areas, the supply of mental health services are less than the urban areas (7). Hospitalization costs, LOS, and frequency of hospitalization were all found to be lower among rural inpatients than among urban inpatients. According to the National Health and Family Planning Commission of China, the consultation rate for mental health services is 0.7% after 2 weeks and the hospitalization rate is 0.5% (8).

There is significant income discrimination among Chinese populations (7). Before 2003 (9), the rural population, which accounts for 60% of the Chinese population, lacked health insurance. Not only are more mental health services excluded from insurance coverage but also the eligible mental health services are often subject to higher co-pays and are capped at a maximum number of covered treatments (10).

The economic crisis during the difficult transition to a market economy in former countries of the Union of Soviet Socialist Republics and Eastern Europe in the 1990s was extremely harmful to both mental and physical health. However, in established market economies, recession crises in which economic growth is zero or negative have been usually found associated with improved physical health, but worse mental health.

Economic conditions affect health outcomes by generating stress; thus, stress connected with low or negative economic growth (e.g., uncertainty, job insecurity, or joblessness) is known to generate psychological distress (such as anxiety and depression symptoms).

Moreover, a healthcare reform that started in the 1980s led to the collapse of the Cooperative Medical Scheme (CMS), a collective economy and prepaid health security system in rural China. Rural healthcare reverted to being primarily privately financed. Most rural residents, who at the time accounted for almost 60% of China's total population, did not have any form of health insurance before 2003.

The Chinese government launched the New Cooperative Medical Scheme (NCMS) in rural China to address such bias (11). After 2012, 97% of rural people and 89% of urban people were covered by one of China's three public health schemes (12).

According to the People's Republic of China's Mental Health Law, medical and pharmaceutical expenditures incurred as a result of serious mental illness are covered by public health insurance (13). There are still reimbursement inequalities based on age, sex, occupation, and other factors (14). Since 2009, hospitalization for mental illness has been covered under China's national public healthcare program (6).

The main disadvantage of China's national health insurance program is that it is not portable (7). In China, the pooling district of the basic medical insurance scheme is based at the county or city level. Therefore, if a rural mental health patient seeks care in a hospital outside the pooling district, he or she will face extremely complex reimbursement procedures and may simply not get reimbursed. Meanwhile, China's healthcare budget is heavily skewed toward physical diseases and urban areas. Most of the large psychiatric hospitals, too, are located in urban areas.

### Reimbursement ratio variable

The reimbursement ratio (RR) was defined as the percentage of costs reimbursed by health insurance over total medical costs (TC). The exact equation is RR = (TC–OOP)/TC ^*^ 100%, where TC represents the total medical costs and OOP represents the out-of-pocket payments made by individuals for their healthcare services. In our sample, the actual RR ranged from 0 to 100%. In addition to insurance type, we used actual RR in this study to measure health insurance. Compared with conventional measures such as insurance type or policy, RR is a more accurate measure in determining how generous the health insurance is in terms of coverage (15, 16).

Rural residents are not able to afford high inpatient costs, due to the low insurance reimbursement level of rural patients. Under the current insurance system, the average RR of rural health insurance is more than 20% lower than that of insurance provided in urban areas and uses significantly less inpatient mental healthcare than those in urban areas. The relatively low cost of outpatient visits in rural areas may encourage people to seek out physicians for consultation but avoid inpatient services. Moreover, the limited availability of specialists in rural areas, as well as the greater travel time and distance involved in seeking care, may contribute to this difference. Therefore, the rural people of China consider mental treatment as an economic burden.

An overview of China's current public health insurance plan (Table 1). The reimbursement ratio under public health insurance schemes is lower in rural insurance schemes than in urban insurance systems (8).

**Table 1:** The overview of the existing public health insurance scheme of China (8).

**Parameters**	**Rural insurance scheme**	**Urban insurance scheme**	
**Name**	**NCMS**	**URBMI**	**UEBMI**
Establishment	2003	2007	1999
Enrollment unit	Household	Household	Individual
Enrollment type	Optional	Optional	Compulsory
Management status	Province	Municipal	Municipal
Management body	National Health and Family Planning Commission of China	Ministry of Human Resources and Social Security	Ministry of Human Resources and Social Security
Enrollees	832,000,000	271,200,000	26,400,000
Financing	410 RMB/person	100–560 RMB/person depending upon a person	The employer
Contributions of government	320 RMB/ person	40–560 RMB/ person depending upon a person	5–7% of the salary of the employee
Purposes	Inpatients and outpatients	Inpatients and outpatients	Inpatients and outpatients
Deductible	300–2,000 RMB	300–900 RMB	400–1,200 RMB
Limit for year	50,000–80,000 RMB	50,000–160,000 RMB	55,000–290,000 RMB
Ratio of reimbursement	30–70%	50–70%	80–100%
Method of payment	Fee-for-service	Fee-for-service	Fee-for-service

The study proves that insurance increases the utilization of health services (17, 18). The authors found that participating in the NCMS improved the use of preventive care but did not increase the utilization of formal medical services. People living in cities of China spend more money on mental illness than those in the countryside of China (19).

There are only some research studies on the use of mental healthcare services by Chinese people. Furthermore, existing research does not address such prejudice. Available research is based on investigated survey metrics on public health services in China's rural and urban locations. However, the hospitalization costs, LOS, and frequency of hospitalization of urban patients were found to be consistently higher than those of rural patients at almost all RR levels. However, when the RR increases, urban–rural differences in the utilization of mental health services significantly decrease.

## Materials and methods

### Ethics approval and consent to participate

As a retrospective study, the designed protocol was waived for registration in the Chinese trial registry and approval (application no. BAnU154x dated 1 January 2021) by the institutional review board. The study follows the law of China and the V2008 Declaration of Helsinki.

### Inclusion criteria

Patients who had taken mental health services for mental disorders from 1 January 2005 to 31 December 2020 were included in the study.

### Exclusion criteria

Patients with incomplete data, more than 1 year of hospital stays, patients with multiple comorbidities, and patients with unclear mental reasons for admission were excluded from the analysis.

### Sociodemographic characteristics

Information regarding age, sex, educational status, marital status, place of residence, etc., was collected from hospital records of the institutes.

### Clinical characteristics

Disease conditions were evaluated based on the International Classification of Diseases, 11th vs. ICD-11 (20). Accordingly, the primary diagnosis codes of mental illness were as follows: F0, organic mental disorders including symptomatic disorders and dementia; F1, mental and behavioral disorders due to the use of psychoactive substances; F2, schizophrenia, schizotypal, and delusional disorders; F3, mood [affective] disorders; F4, neurotic, stress-related, and somatoform disorders; F5, behavioral syndromes associated with physiological disturbances and physical factors; F6, disorders of adult personality and behavior; F7, mental retardation; and F8–9, others.

### Cost-related information

The cost of therapies, diagnostic costs, and hospitalization costs was evaluated.

### Insurance information

Insurance covered, nature of insurance, amount reimbursed, and out-of-pocket costs at the time of discharge were evaluated.

### Reimbursement ratio variable

The percentage of costs that were reimbursed by the health insurance of an individual over total medical costs spent by the individual is the reimbursement ratio. The reimbursement ratio was calculated as per equation (1) (15):


(1) $$\begin{array}{l} \;Reimbursement\;ratio\\ =\;\frac{Total\;medical\;cost-Out\;of\;the\;pocket\;payment}{\;total\;medical\;cost}\\ \;\begin{array}{l} \times 100 \end{array} \end{array}$$

### Frequency of hospitalization

Many patients were admitted to hospitals because of severe types of mental disorders. Therefore, the frequency of hospitalization was evaluated.

### Mental health inpatient services preference

Mental health services and clinical parameters of patients were described using descriptive analysis. The length of hospitalization, frequency of hospitalization, and hospitalization costs were used to evaluate mental health inpatient services preference (6, 19).

### Disparities in the utilization of mental health services

Disparities in the utilization of mental health services were evaluated by models 1 and model 2 through equations (2) and (3), respectively (7):


(2) $$\begin{array}{l} \begin{array}{lll} Utilization & = & \alpha +\;\beta_{1}\times X\;+\;\beta_{2}\times Insurance\;+\;\beta_{3} \end{array}\\ \;\times \;Reimbursement\;ratio\;+\;e \end{array}$$

(3) $$\begin{array}{l} \begin{array}{lll} Utilization & = & \alpha +\;\beta_{1}\times X\;+\;\beta_{2}\times Insurance\;+\;\beta_{3} \end{array}\\ \;\times \;Reimbursement\;ratio\;+\;\beta_{4}\\ \;\begin{array}{l} \times (Reimbursement\;ratio\;\times \;insurance)+\;e \end{array} \end{array}$$

where X indicates the sociodemographic and clinical characteristics.

The coding of sociodemographic and clinical characteristics is presented in Table 2.

**Table 2:** The coding of sociodemographic and clinical characteristics.

**Characteristics**	**Code value**
Municipal hospital	1
Provincial hospital	2
Male	1
Female	2
Age (years)	Actual values
Married	1
Single	2
Urban	1
Rural	2
Schizophrenia	1
Depression	2
Other	3
Stable condition	1
Severe condition	2
Critical condition	3
NCMS insurance	1
URBMI	2
UEBMI	3
Reimbursement ratio	Actual values

## Statistical analyses

GraphPad software (San Diego, USA) employed InStat 3.01 for statistical analysis. For categorical data statistical analysis, the Mann–Whitney *U*-test was utilized. Multiple regression analysis coupled with the Poisson model was used to assess the influence of health insurance on the usage of mental healthcare. The Mann–Whitney *U*-test was used to compare the usage of mental health services in urban and rural locations. For the distribution of hospitalization duration and expenditures, log transformation was utilized. A *p*-value of < 0.05 was regarded as significant.

## Results

### Study population

**Figure 1:**
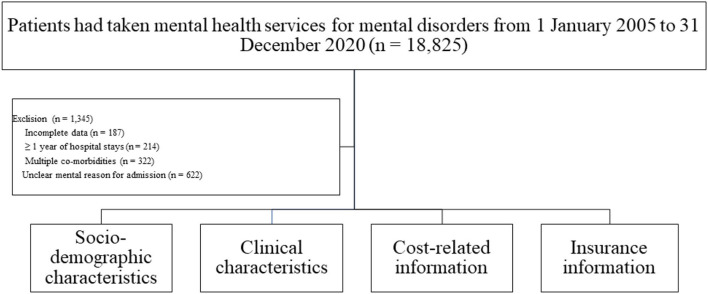
The flow diagram of the study.

From 1 January 2005 to 31 December 2020, a total of 18,825 patients received mental healthcare for mental illnesses at the parent and referral institutions. Among them, 187 had missing data, 214 had more than 1 year of hospital stays, 322 had numerous comorbidities, and 622 had an unclear mental cause for admission. As a result, data from these patients (*n* = 1,345) were not included in the study. The investigation includes data on 17,480 patients' sociodemographic and clinical features, cost-related information, and insurance information. The flow diagram of the study is shown in Figure 1.

### Sociodemographic characteristics

A total of 9,811 males (56%) and 7,669 females (44%) were admitted to mental health services for mental illnesses. The majority of the 6,743 patients (39%) were between the ages of 40 and 60 years. The majority of the admitted patients were single. The majority of the patients had a low level of education. Table 3 shows the information on the sociodemographic features of the enrolled patients.

**Table 3:** Sociodemographic characteristics of enrolled patients.

**Characteristics**	**Value (%)**
Total number of patients included in the analysis	17,480
**Nature of hospital**	
Municipal	6,205 (35)
Provincial	11,275 (65)
**Sex**	
Male	9,811 (56)
Female	7,669 (44)
**Age (years)**	
≤ 39	5,069 (29)
40–60	6,743 (39)
≥61	5,668 (32)
Mean ± SD	45.24 ± 8.52
**Marital status**	
Married	5,598 (32)
Single	11,882 (68)
**Registered place of residence**	
Urban	9,845 (56)
Rural	7,635 (44)
**Ethnicity**	
Han Chinese	14,754 (84)
Mongolian	2,021 (12)
Tibetan	503 (3)
Uighur Muslim	202 (1)
**Education status**	
Very primitive	7,241 (41)
Primitive	4,322 (25)
School level	3,245 (19)
Educate but below graduate	1,942 (11)
Graduate and above	730 (4)

### Clinical characteristics

Most patients were diagnosed with schizophrenia followed by depression and stable conditions. The details of the diagnostic condition of the enrolled patients are reported in Table 4.

**Table 4:** Clinical characteristics of the enrolled patients.

**Characteristics**	**Value (%)**
Total number of patients included in the analysis	17,480
Diagnosis	
Schizophrenia	10,345 (59)
Depression	4,370 (25)
Other	2,765 (16)
Severity	
Stable condition	10,813 (62)
Severe condition	4,513 (26)
Critical condition	2,154 (12)
Length of hospitalization	
Minimum (days)	5
Maximum (days)	85
Mean ± SD (days)	58.45 ± 12.11
Frequency of hospitalization	
Minimum	1
Maximum	5
Mean ± SD	1.58 ± 0.24

### Cost-related information

The cost of therapies was the highest followed by hospitalization costs and diagnostic costs. The value of the different costs of the enrolled patients is reported in Table 5.

**Table 5:** Cost-related information of the enrolled patients.

**Characteristics**	**Value (mean ±SD)**
Total number of patients included in the analysis	17,480
Cost of therapies/patient/year	17,812 RMB
Diagnostic costs/patient/year	4,312 RMB
Hospitalization cost/patient/year	13,452RMB

### Insurance information

A total of 14,452 (83%) patients had insured insurance, while 3,028 (17%) did not. The health insurance policy could compensate 10,732 (62%) of the 14,452 patients who had insured an insurance policy. However, 3,720 patients (21%) did not obtain reimbursements from their health insurance policies for one or more reasons (e.g., service had been taken outside the state, and low insurance). At the time of discharge, the majority of patients had to pay out-of-pocket expenses. Table 6 displays the specifics of the registered patients' insurance information.

**Table 6:** Insurance information of the enrolled patients.

**Characteristics**	**Value (%)**
Total number of patients included in the analysis	17,480
Insurance covered	
Yes	14,452 (83)
No	3,028 (17)
Amount reimbursed	
Yes	10,732 (62)
No	3,720 (21)
Nature of insurance	
URBMI	7,643 (44)
UEBMI	1,358 (8)
NCMS	5,451 (31)
Out-of-pocket costs at the time of discharge	
Yes	17,012 (97)
No	468 (3)
Value (Mean ± SD; RMB)/patient/year	2,012

### Reimbursement ratio variable

The reimbursement ratio variable was 64.12 ± 8.45% (minimum 50 %; maximum 75%).

### Mental health inpatient services preference

The length of hospitalization (*p* < 0.0001), frequency of hospitalization (*p* = 0.006), and hospitalization costs (*p* = 0.001) of urban patients were high than those of rural patients. The details of mental health inpatient services preference disparities in the utilization according to a registered place of residence are presented in Table 7.

**Table 7:** Mental health inpatient services preference disparities in the utilization according to a registered place of residence.

**Parameters used to evaluate mental** **health inpatient services preference**	**Registered place of residence** **[value (%)]**		**Comparisons**
	**Urban**	**Rural**	
Numbers of patients	9,845	7,635	*p*-value
Length of hospitalization	65 ± 11 days	33 ± 8 days	< 0.0001
Frequency of hospitalization	3.12 ± 0.45	1.55 ± 0.21	0.006
Hospitalization costs	14,041 ± 1,054 RMB	10,951 ± 542 RMB	0.001

The Mann–Whitney *U*-test was used for the evaluation of differences.

A *p*-value < 0.05 was considered significant.

Patients who covered insurance had a higher length of hospitalization (*p* < 0.0001), frequency of hospitalization (*p* < 0.0001), and hospitalization costs (*p* < 0.0001) than those who did not cover insurance. The details of mental health inpatient services preference disparities in the utilization according to covered insurance are presented in Table 8.

**Table 8:** Mental health inpatient services preference disparities in the utilization according to covered insurance.

**Parameters used to evaluate mental** **health inpatient services preference**	**Covered insurance [value (%)]**		**Comparisons**
	**Yes**	**No**	
Numbers of patients	14,452	3,028	*p*-value
Length of hospitalization	73 ± 12 days	17 ± 8 days	< 0.0001
Frequency of hospitalization	3.85 ± 1.11	1.35 ± 0.15	< 0.0001
Hospitalization costs (RMB)	18,985 ± 1,245	8,542 ± 980	< 0.0001

The Mann-Whitney *U*-test was used for the evaluation of differences.

A *p*-value < 0.05 was considered significant.

Patients who covered insurance through the URBMI health insurance scheme had the longest hospitalizations, the most frequent hospitalizations, and the highest hospitalization costs, followed by patients who covered insurance through the UEBMI health insurance scheme and patients who covered insurance through the NCMS health insurance scheme. Table 9 shows the specifics of mental health inpatient service preference discrepancies in usage based on the kind of insurance.

**Table 9:** Mental health inpatient services preference disparities in the utilization according to the nature of insurance.

**Parameters used to evaluate mental health inpatient services preference**	**Covered insurance [value (%)]**			**Comparisons**
	**URBMI**	**UEBMI**	**NCMS**	
Numbers of patients	7,643	1,358	5,451	*p*-value
Length of hospitalization	70 ± 13 days	69 ± 6 days	25 ± 7 days	< 0.0001
Frequency of hospitalization	3.11 ± 1.25	2.45 ± 0.85	1.85 ± 0.14	< 0.0001
Hospitalization costs (RMB)	17,841 ± 2,452	11,541 ± 1,844	7,845 ± 560	< 0.0001

The Mann–Whitney *U*-test was used for the evaluation of differences.

A *p*-value < 0.05 was considered significant.

**Figure 2:**
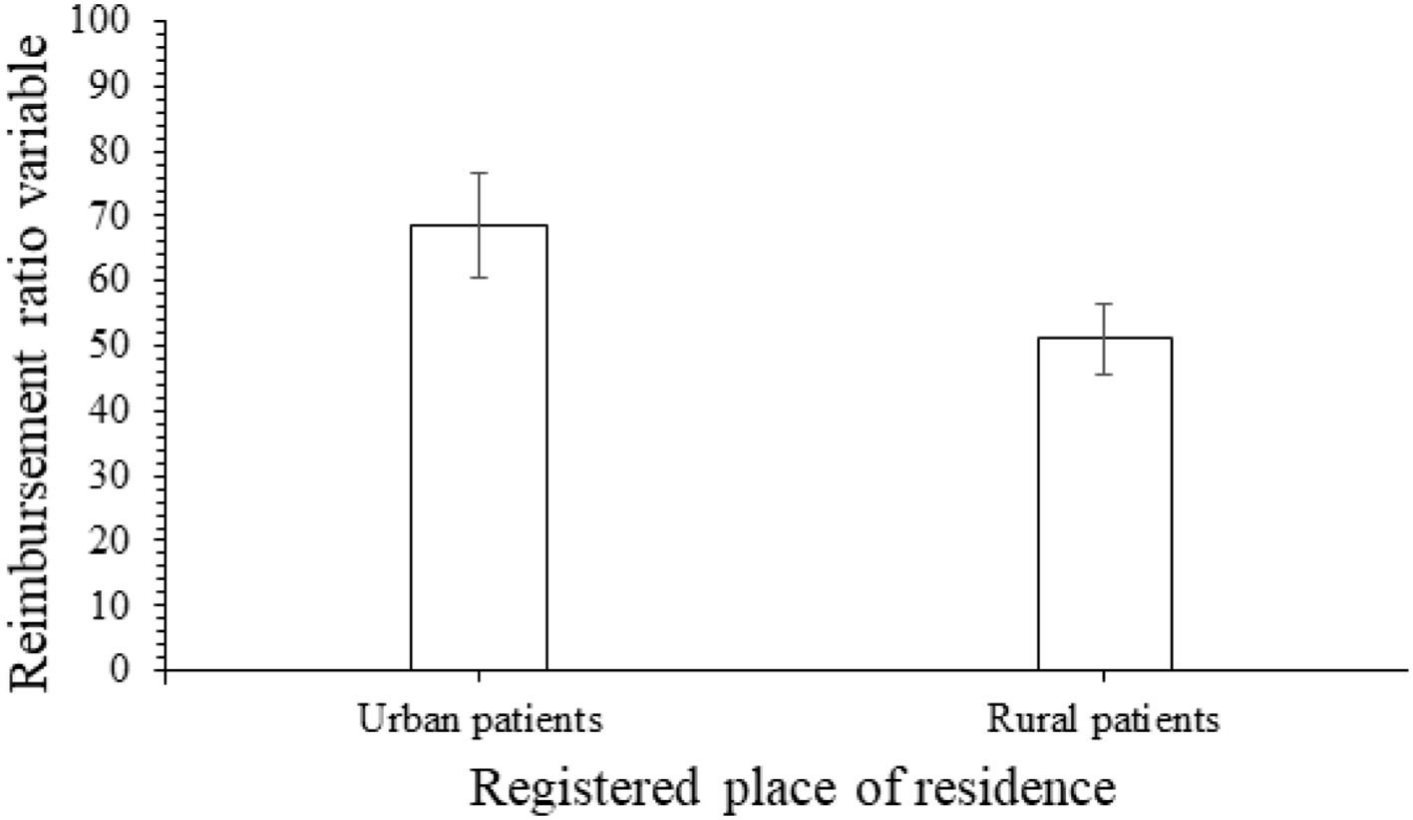
The reimbursement ratio varies according to the registered place of residence.

**Figure 3:**
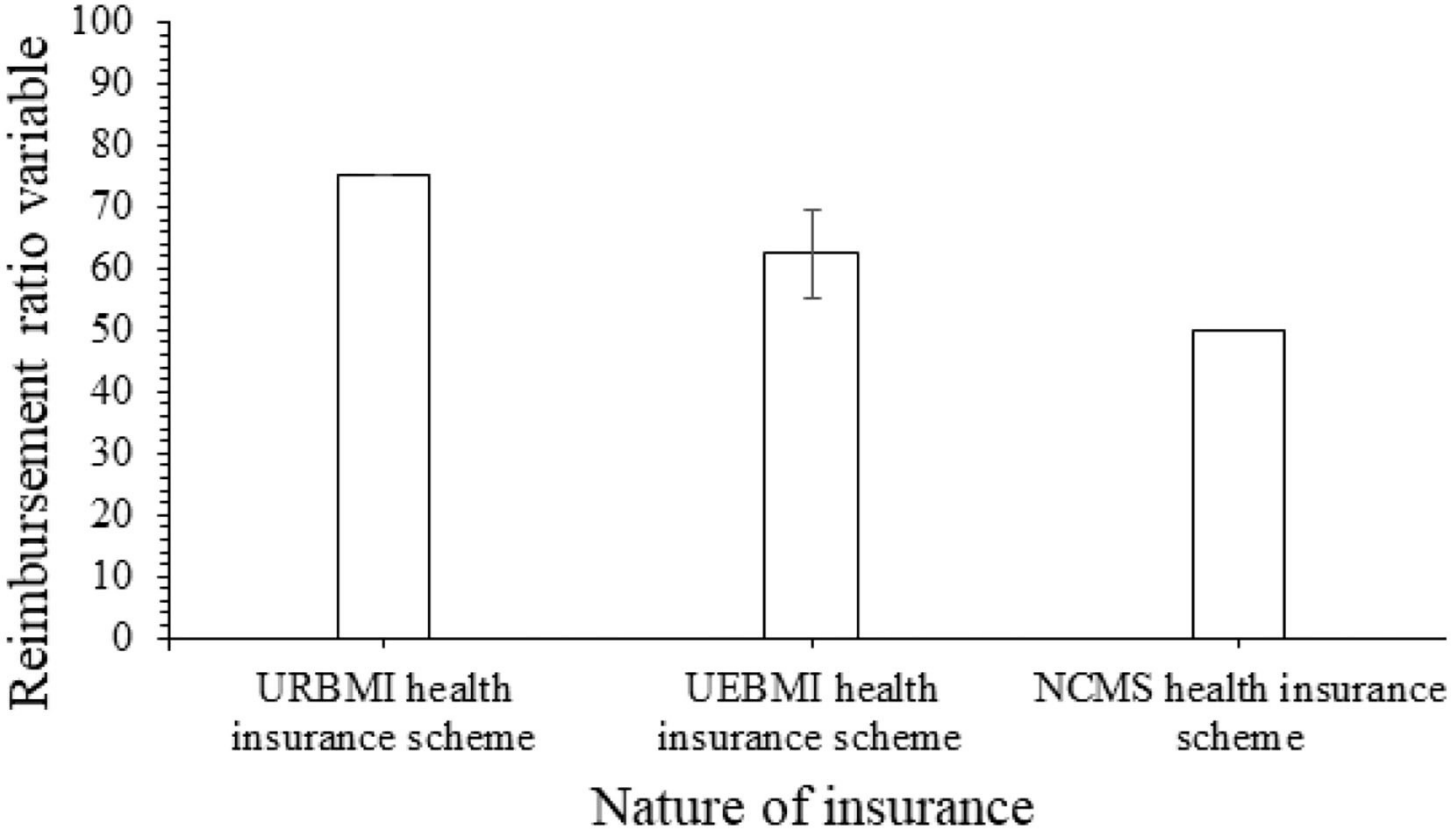
The reimbursement ratio varies according to the nature of the insurance.

Urban patients had a greater reimbursement ratio variable than rural patients (68.52, 8.15% vs. 51.15, 5.45%, *p* = 0.0001, Figure 2). Furthermore, the reimbursement ratio variable was higher for patients who covered insurance through the URBMI health insurance scheme (75%), followed by patients who covered insurance through the UEBMI health insurance scheme (62.5 7.14%) and patients who covered insurance through the NCMS health insurance scheme (50%, Figure 3).

### Disparities in the utilization of mental health services

Urban residents used mental health services more than rural residents. Furthermore, when the reimbursement ratio variable was larger, consumers preferred mental healthcare, particularly in rural areas. Table 10 shows the findings of models 1 and 2 for multiple regression analysis of inequalities in the consumption of mental healthcare.

**Table 10:** Multiple regression analysis for disparities in the utilization of mental health services.

**Variables**	**Length of**		**Frequency of**		**Hospitalization costs**	
	**hospitalization**		**hospitalization**		**(RMB)**	
	**Model 1**	**Model 2**	**Model 1**	**Model 2**	**Model 1**	**Model 2**
Nature of hospital	0.061	0.065	0.058	0.059	0.042	0.043
Sex	0.059	0.062	0.058	0.064	0.065	0.062
Age (years)	0.052	0.053	0.049	0.048	0.065	0.063
Marital status	0.058	0.059	0.061	0.062	0.063	0.064
Registered place of residence	0.023	0.024	0.026	0.038	0.041	0.042
Diagnosis	0.029	0.035	0.041	0.042	0.045	0.046
Severity	0.042	0.043	0.041	0.041	0.042	0.043
Nature of insurance	0.041	0.041	0.042	0.043	0.042	0.042
Reimbursement ratio variable	0.045	0.046	0.045	0.046	0.047	0.048

A *p*-value < 0.05 was considered significant.

## Discussion

Equal access to healthcare is an important mechanism that mediates socioeconomic disparities in people's health including mental well-being. A growing number of studies consistently show that people from higher socioeconomic groups are more likely to experience better health outcomes, report a lower risk of chronic conditions, and enjoy a longer life expectancy than those with lower socioeconomic status (SES). While looking specifically at rural populations, who have less utilization of or more unmet needs regarding medical care, a similar pattern emerges. Despite significant policy attention and years of study, equal access to healthcare services in rural settings remains a health policy challenge.

Because of poor financial backup, most individuals did not prefer physical and/or more mental treatment(s), owing to the high expense of treatments, particularly in rural China (21). In addition, traditional Chinese religious beliefs and superstitions are used to treat patients suffering from delusions and hallucinations in China (22). Rural Chinese people believe that mental health treatments are an economic burden.

According to the research, people with mental problems in rural regions opted less for mental health treatments than those in metropolitan areas. One probable explanation is that (23) outpatient expenses are lower than inpatient expenses, particularly in rural uninsured populations. In addition, facilities for indoor patients are few in rural areas. The relatively low cost of outpatient visits in rural areas may encourage people to seek out physicians for consultation but avoid inpatient services. Moreover, the limited availability of specialists in rural areas, as well as the greater travel time and distance involved in seeking care, may contribute to this difference. Because medical insurance reimbursement limitations are lower in rural locations, many cannot afford to pay hefty inpatient expenses. A Chinese individual living in an urban region has a disposable income of 10,309 RMB per capita, whereas a person living in a rural location has a disposable income of 5,084 RMB (24). For the creation of health insurance plans for rural residents, uniform steps must be done.

To reduce these disparities, the Chinese government implemented the New Cooperative Medical Scheme (NCMS) among the rural population in 2003 (11). As for urban residents, previous public health insurance schemes were integrated into the Urban Employee Basic Medical Insurance (UEBMI) and the Urban Resident Basic Medical Insurance (URBMI) (12). By the end of 2012, 89% of urban residents and 97% of rural residents were covered by one of the country's three main public health insurance schemes, namely UEBMI, URBMI, and NCMS, up from only 55% of urban residents and 21% of rural residents insured in 2003 (8).

As of 2009, severe mental illnesses were incorporated into the national public health service program (6). In 2012, the Mental Health Law of the People's Republic of China was passed. Under this law, medical expenses for mental illnesses, just like common illnesses, should be covered by the three basic types of public health insurance.

However, there are large variations in the actual reimbursement ratios (RRs) for mental healthcare, partly depending on the type of health insurance. Even for people covered by the same insurance scheme, coverage could still be different based on factors such as age, sector of work, and whether they are retired or not.

Patients who had insurance used more mental health treatments than those who did not have insurance. Most Chinese individuals cannot afford the exorbitant treatment costs (25). The present research indicates that even if the insurance program is implemented in rural China, the reimbursement ratio variable is lower (26). Socioeconomic variables influence rural Chinese healthcare utilization (15). The NCMS is insufficient to alleviate the Chinese people's economic burden.

According to the analysis, just 63% of the registered people would get compensation. In China, the health insurance plan is not portable (7). China's healthcare expenditure is biased toward metropolitan regions and physical diseases (27). Furthermore, significant mental facilities are located in cities. The existing Chinese healthcare system is a barrier to compensation for mental health treatments.

The research has significant limitations, including the use of solely inpatient data for analysis. Our data do not provide information on outpatient and community mental health services, which are quite important in China, particularly considering China's widespread “free medication” program (“686” program) that provides a considerable amount of mental health services to people with severe mental disorders at the community level (28–32).

Also, the patients with private insurance were barred from participating in the trial because private insurance in China is still nascent, mainly targeting high-income households and covering only about 7% of the population (33).

The data used were from the record books of two large mental health hospitals in Shandong Province, and the sample may not be representative of the entire country.

## Conclusion

The expansion of public health insurance has been an important item on the healthcare reform agenda in China. To date, the country's public health insurance covers more than 90% of its citizens. But reform should also address issues such as the wide variation in coverage across different types of insurance, particularly between urban and rural areas.

Uniform measures are required to be taken for the development of health insurance policies for people in rural areas. The NCMS is not enough to reduce the economic burden of the Chinese population. The current health system scheme of China is a barrier to reimbursements for the utilization of mental health services.

The study suggests that the Chinese government should raise reimbursement ratio variables in instances of the NCMS health insurance plan to promote rural mental health.

However, a small-scale increase in the RR of the NCMS is less likely to result in an improvement in the utilization of rural mental health services. As discussed earlier, the current co-payment rate under the UEBMI is about 80%, significantly higher than that under the NCMS (about 30%).

To reduce rural–urban disparities in healthcare utilization, the Chinese government launched the gradual merger of the URBMI and NCMS systems in 2016, to establish a uniform basic medical insurance system for rural and urban residents. During this process, policymakers in China would need to consider significantly improving insurance coverage (particularly in rural areas), including increasing the RR and the annual reimbursement limit and expanding coverage services to mental inpatients to cover the unmet need (4).

Severe mental illnesses have now been classified as, simply, severe illness, which implies that patients with severe mental disease can apply for a higher reimbursement if they receive treatment in the local, designated hospital. However, as we discussed earlier, limited specialists and the limited portability of health insurance pose serious obstacles to rural patients.

Health insurance coverage, particularly the reimbursement ratio, could be a powerful policy tool to influence people's healthcare utilization. To improve access to and reduce rural–urban disparities in mental healthcare, future healthcare reform in China should consider expanding mental health coverage, particularly in rural areas, as well as making health insurance portable.

## Data availability statement

The original contributions presented in the study are included in the article/supplementary material, further inquiries can be directed to the corresponding author.

## Ethics statement

The studies involving human participants were reviewed and approved by as a retrospective study, the designed protocol was waived for registration in the Chinese trial registry and approval (Application No. BAnU154x dated 1 January 2021) by the institutional review board. The study follows the law of China and the V2008 Declarations of Helsinki. The patients/participants provided their written informed consent to participate in this study.

## Author contributions

YY: aim and objective of the study, collection of data, assessment of data, writing, and submission. YT: revisions of the paper, critical assessment of the paper, and resubmission. Both authors contributed to the article and approved the submitted version.
